# Aurora Kinase A as a Diagnostic and Prognostic Marker of Malignant Mesothelioma

**DOI:** 10.3389/fonc.2021.789244

**Published:** 2021-12-08

**Authors:** Zhenying Guo, Li Shen, Ningning Li, Xiaoxiao Wu, Canming Wang, Zheng Gu, Zhongjian Chen, Junping Liu, Weimin Mao, Yuchen Han

**Affiliations:** ^1^ Department of Pathology, Shanghai Chest Hospital, Shanghai Jiao Tong University, Shanghai, China; ^2^ School of Medicine, Shanghai Jiao Tong University, Shanghai, China; ^3^ Department of Pathology, Cancer Hospital of the University of Chinese Academy of Sciences (Zhejiang Cancer Hospital), Hangzhou, China; ^4^ Institute of Basic Medicine and Cancer, Chinese Academy of Sciences, Hangzhou, China; ^5^ Zhejiang Key Laboratory of Diagnosis and Treatment Technology on Thoracic Oncology, Zhejiang Cancer Hospital, Hangzhou, China; ^6^ Office of Education, Sir Run Run Shaw Hospital, Zhejiang University School of Medicine, Hangzhou, China; ^7^ Department of Clinical Medicine Engineering, Second Affiliated Hospital of Zhejiang University, School of Medicine, Hangzhou, China; ^8^ Cancer Hospital of University of Chinese Academy of Sciences, Zhejiang Cancer Hospital, Hangzhou, China

**Keywords:** malignant mesothelioma, bioinformatics, biomarkers, tissue microarray, Aurora kinase A

## Abstract

**Background:**

Malignant mesothelioma (MM) is a highly aggressive cancer with a poor prognosis. Despite the use of several well-known markers, the diagnosis of MM is still challenging in some cases. we applied bioinformatics to identify key genes and screen for diagnostic and prognostic markers of MM.

**Methods:**

The expression profiles of GSE2549 and GSE112154 microarray datasets from the Gene Expression Omnibus database contained 87 cases of MM tissue and 8 cases of normal mesothelial tissue in total. The GEO2R tool was used to detect differentially expressed genes (DEGs). Gene ontology (GO) and Kyoto Encyclopedia of Genes and Genomes (KEGG) pathway enrichment analyses of DEGs were performed using DAVID Bioinformatics Resources. The DEGs protein-protein interaction networks were constructed from the STRING database. Cytoscape was used to identify significant modules and hub genes. The GEPIA database was used to explore relationships between hub genes and prognosis of MM. Immunohistochemistry was used to analyze protein expression in tissue microarrays with 47 Chinese MM tissues. Statistical analyses diagnostic and prognostic values.

**Results:**

346 DEGs were identified: 111 genes upregulated, and 235 downregulated. GO analysis showed that the primary biological processes of these DEGs were cell adhesion, leukocyte migration, and angiogenesis. The main cellular components included the extracellular space, extracellular exosome, and extracellular region. The molecular functions were integrin binding, heparin binding, and calcium ion binding. KEGG pathway analysis showed that DEGs are primarily involved in PPAR signaling pathway, extracellular matrix–receptor interactions, and regulation of lipolysis in adipocytes. Survival analysis showed that seven genes—*AURKA, GAPDH, TOP2A, PPARG, SCD, FABP4*, and *CEBPA*—may be potential prognostic markers for MM. Immunohistochemical studies showed that Aurora kinase A (*AURKA* gene encode, Aurora-A) and GAPDH were highly expressed in MM tissue in comparison with normal mesothelial tissue. Kaplan-Meier analysis confirmed a correlation between Aurora-A protein expression and overall survival but did not confirm a correlation with GAPDH. The receiver operating characteristic curves of Aurora-A protein expression suggested acceptable accuracy (AUC = 0.827; 95% CI [0.6686 to 0.9535]; *p *= 0.04). The sensitivity and specificity of Aurora-A were 83.33% and 77.78%, respectively.

**Conclusion:**

Aurora-A could be an optimal diagnostic biomarker and a potential prognostic marker for MM.

## Introduction

Malignant mesothelioma (MM) is an aggressive cancer that arises from the mesothelial cells lining the pleura, peritoneum, pericardium, and tunica vaginalis. MM primarily affects older people who have been occupationally exposed to asbestos (1). The median survival of patients with MM is less than one year, and their 5-year survival rate is less than 5%. Although the development of multidisciplinary treatment, including surgical resection, radiotherapy, chemotherapy and targeted immunotherapy, the diagnosis and treatment of malignant mesothelioma is still a big challenge. Therefore, it is important to identify the diagnostic and prognostic value of markers that can aid in selecting patients who will benefit from treatments (2).

Genomic studies have been conducted on pleural MM subsets using microarray-based comparative genomic hybridization, fluorescence *in situ* hybridization, and targeted DNA sequencing with the ultimate goal of finding new molecular targets. genetic alterations (*BAP1*, *NF2*, *TP53*, and *CDKN2A* mutations and/or copy number alterations) commonly found in pleural mesothelioma are also present in diffuse peritoneal MM, although their frequencies vary according to the anatomical localization (3–5).

Integrating and reanalyzing these genomic data offer possibilities for identifying specific disease-related biomarkers. Recently, two studies used bioinformatics methods to analyze MM microarrays based on the Gene Expression Omnibus (GEO) dataset GSE51024 for the identification of differentially expressed genes (DEGs) between pleural MM tissues and normal lung tissues (6, 7). However, MM is derived from mesothelial cells on the surface of the pleura and peritoneum. Therefore, using pleural or peritoneal tissue as a negative control can obtain more accurate data. In this study, we identified robust and stable DEGs between MM tissues and normal pleural or peritoneal tissues from GEO microarray datasets and assessed potential functions of the genes. Seven hub genes were selected with prognostic value for MM. Subsequently, tissue microarray (TMA) was used to validate protein expression encoded by DEGs and evaluate the diagnostic and prognostic value. Finally, Aurora-A was identified as an optimal diagnostic biomarker and a potential prognostic marker for MM. In conclusion, these results provide a novel biomarker for diagnosis and prognosis, as well as potential therapeutic targets for MM.

## Material And Methods

### Microarray Data

Using the keywords “malignant mesothelioma [Accession]” to search the GEO database (https://www.ncbi.nlm.nih.gov/geo/), we downloaded the gene expression profiles of GSE2549 (8, 9) and GSE112154 (10). The platform for GSE2549 is GPL96 Affymetrix Human Genome U133A Array (HG-U133A), which includes pleural MM surgical tissues (n = 42), normal pleura tissues (n = 5). The platform for GSE112154 is GPL10558 Illumina HumanHT-12 V4.0 expression bead chip, which includes 45 frozen surgical tissues of diffuse malignant peritoneal mesothelioma, 3 normal peritoneal tissues. The dataset information is shown in **Table 1**. In addition, MM RNA-sequencing and clinical data were downloaded from The Cancer Genome Atlas database (https://cancergenome.nih.gov/) and used in the study.

**Table 1:** Characteristics of the included datasets.

GEO Dataset ID	GSE2549	GSE112154
Platform	GPL96	GPL10558
Number of Rows per platform	22283	48107
Country	USA	Italy
Tumor Site	Pleura	Peritoneum
Number of samples		
Tumor tissue	42	45
Normal pleural mesothelium	5	/
Normal peritoneal mesothelium	/	3
Reference	Gordon GJ et al. (8)	Sciarrillo R et al. (10)

### DEGs Screening

The GEO2R tool (https://www.ncbi.nlm.nih.gov/geo/geo2r) was used to detect DEGs between MM and normal pleural or peritoneal tissues. the cut-off criteria was (*p* < 0.01 and |logFC| ≥ 1). Statistical analysis was carried out for each dataset, and the intersecting part was identified using the Venn diagram web tool (bioinformatics.psb.ugent.be/webtools/Venn). ﻿Heatmap was drawn on the normalized expression matrix using the pheatmap package in R software Volcano plot was drawn using the ggplot2 package in R software

### Functional DEGs Enrichment Analyses

Gene ontology (GO) functional annotations and Kyoto Encyclopedia of Genes and Genomes (KEGG) pathway analysis of DEGs were performed using the DAVID Bioinformatics Resources 6.8 database (https://david.ncifcrf.gov/home.jsp).

﻿ Gene set enrichment analysis (GSEA) was performed using Gene Set Enrichment Analysis (GSEA, version 4.1.0, http://www.gsea-msigdb.org/gsea/index.jsp) to reveal the critical signaling pathways involving MM.

### Construction and Module Analysis of PPI Network

The STRING database (version 11.0, http://string-db.org) was used to build a protein-protein interaction (PPI) network. a comprehensive Gt score greater than 0.4 was considered statistically significant. Analysis of functional interactions between proteins can be helpful for understanding mechanisms related to disease occurrence and development. Cytoscape (version 3.8.2) was used to visualize and analyze the interaction network. The most important module in the PPI network was identified by the MCODE of Cytoscape. The selection criteria were as follows: MCODE score greater than 5, cut-off value of 2, node cut-off value of 0.2, maximum depth of 100, and k-score of 2.

### Identification of Hub Genes

The CytoHubba plugin of the Cytoscape software was used to select the hub genes and can predict and explore important nodes and subnetworks using several topological algorithms. The Maximal Clique Centrality algorithm was selected to explore hub genes.

### Validation and Survival Analysis of Hub Genes

The GEPIA database (http://gepia.cancer-pku.cn) includes 9736 tumor and 8587 normal tissue samples from The Cancer Genome Atlas database (TCGA) and the Genotype-Tissue Expression (GTEx) projects. Initial survival analysis was conducted with the gene expression profiles and clinical information of 87 MM patients who were diagnosed between 1999 and 2013 in the GEPIA database. Single-gene analysis was used to create plots in MM and normal mesothelium. The threshold was selected as the default value.

### Patient Material

**﻿** Patients diagnosed with MM between 2002 and 2020 in the Cancer Hospital of the University of Chinese Academy of Sciences (Zhejiang Cancer Hospital) were selected. Corresponding H&E slides and immunochemistry slides were reviewed by three pathologists (Z.Guo, N.Li and Y.Han) for confirmation of the diagnosis. Clinical records were accessed: sex, age at diagnosis, family and personal history of cancer, asbestos exposure, lymph node or systemic metastases, and the date of last follow-up or death. Data on histologic type, the presence of vascular invasion and lymph node metastases was extracted from the surgical pathology reports. Follow-up time was calculated as months between the date of the diagnosis and the date of the last follow-up. Patients were considered to be lost to follow-up if ﻿no medical information was obtainable beyond the original surgery. The Institutional Review Board at the Cancer Hospital of the University of Chinese Academy of Sciences (Zhejiang Cancer Hospital) approved this study. 10 normal peritoneal mesothelium (NP) were obtained from patients who underwent surgery for gastric or colon carcinomas as control.

### Tissue Microarray

Formalin-fixed, paraffin-embedded tissue blocks of 47 MM specimens were selected according to tissue availability for the construction of TMA. The TMA consisted of triplicate 1.0-mm cores made using a manual tissue microarray (Beecher Instrument, Sun Prairie, WI, USA). The selection of regions in the tissue block for the cores included two cores from the periphery of the lesion and one core from the center of the lesion. Sections of 4 μm were cut for subsequent immunohistochemical analysis.

### Immunohistochemical Staining

Immunohistochemical staining of the TMA was conducted with the following antibodies: anti-SCD1 (﻿1:250 dilution; Abcam, CD.E10, ab19862), anti-FABP4 (1:10000 dilution; Abcam, EPR3580, ab92501), anti-topoisomerase II alpha (1:5000 dilution; Abcam, EP1102Y, ab52934), anti-C/EBPα (1:200 dilution; CST, D56F10, No. 8178), anti-phospho-Aurora-A (Thr288) (1:800 dilution; CST, C39D8, No. 3079), anti-Aurora-A (1:200 dilution; Abcam, 35C1, ab13824), anti-PPARγ (1:250 dilution; Santa Cruz Biotechnology, E-8, sc-7273), anti-GAPDH (1:800 dilution; CST, 14C10, No. 2118). Negative control slides consisted of substituting normal serum for the primary antibody. A whole section of a MM was used as a positive control. Immunohistochemical staining was scored semi-quantitatively by three pathologists (Z.Guo, N.Li, Y.Han), which is obtained by multiplying the proportion of cells showing staining and the intensity of staining (0, no staining; 1, weak; 2, moderate; and 3, strong). A case was considered to show positive staining when at least 1% of tumor cells showed staining.

### Statistical Analysis

Immunohistochemical data between the MM group and NP group were compared using the Mann-Whitney *U* test. The Kaplan-Meier method was used to estimate survival. The receiver operating curve (ROC) was used to test for diagnostic factors. All statistical analyses were performed using Prism (version 9.2; GraphPad Inc.). A *p* value (two-tail) of less than 0.05 was considered to be statistically significant.

## Results

### Identification and Functional Enrichment Analysis of DEGs in MM

**Figure 1:**
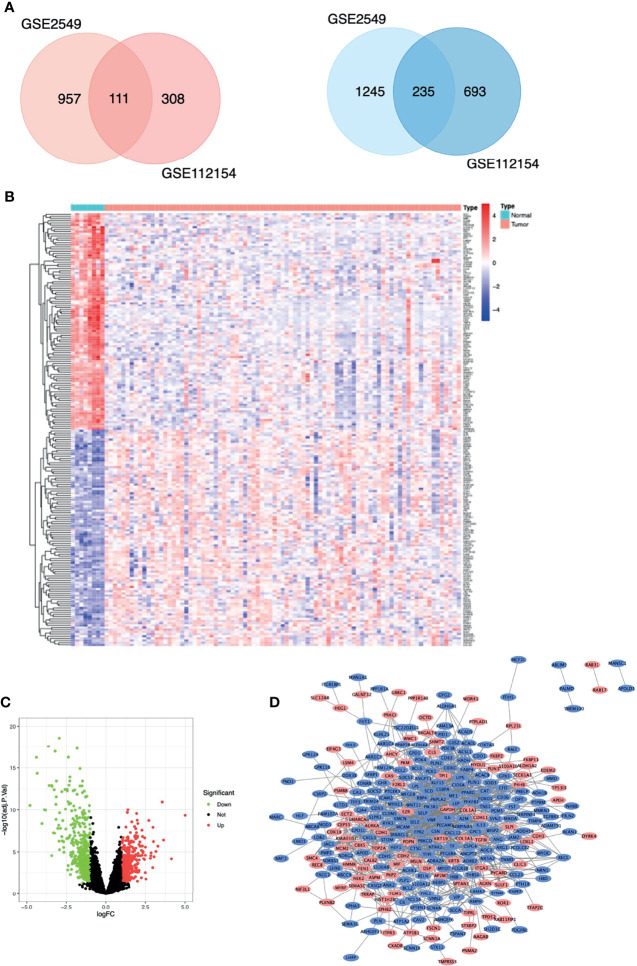
Differentially expressed genes (DEGs) in patients with malignant mesothelioma. **(A)** Venn diagram of DEGs among the mRNA expression profiling sets GSE2549 and GSE112154. Blue denotes downregulated genes, and red denotes upregulated genes showed. **(B)** Hierarchical heat map of TOP100 DEGs between malignant mesothelioma and normal mesothelial tissues. **(C)** Volcano plot of all DEGs between malignant mesothelioma and normal mesothelial tissues. **(D)** Protein-protein interaction network constructed with DEGs using Cytoscape. Red nodes represent upregulated genes, and blue nodes represent downregulated genes.

The MM expression microarray datasets GSE2549 and GSE112154 were used in this study. GSE2549 contained 42 pleural MM samples and 5 normal pleural tissues, and GSE112154 included 45 peritoneal MM tissues and 3 normal peritoneal tissues (**Table 1**). Overall, 346 DEGs were identified: 111 upregulated genes and 235 downregulated genes (**Figure 1A**). Hierarchical heatmap revealed distinct clusters of MM and Normal mesothelial tissues (**Figure 1B**). Volcano plot displayed significantly downregulated (green) or upregulated (red) genes (**Figure 1C**).

**Figure 2:**
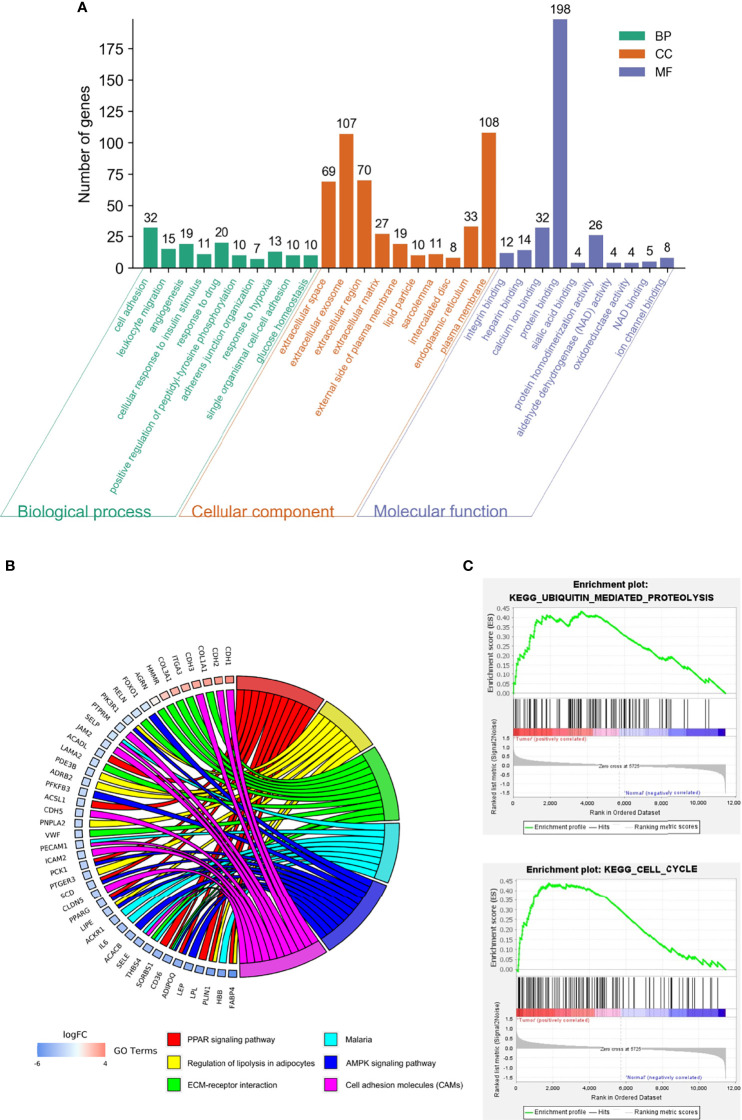
Functional analysis of differentially expressed genes (DEGs). **(A)** Gene ontology (GO) function and Kyoto Encyclopedia of Genes and Genomes (KEGG) pathway enrichment analysis for DEGs were performed using DAVID Bioinformatics Resources. The enriched GO terms were divided into CC, BP, and MF ontologies. **(B)** Chord plot depicting the relationship between genes and GO terms of the top 100 DEGs. **(C)** Gene set enrichment analysis (﻿GSEA) analysis of DEGs.

Biological annotation of the DEGs in MM was performed using the DAVID online analysis tool, and the 346 DEGs were chosen to perform GO and KEGG analyses (**Figure 2**). We detected enrichment in biological process (BP) GO terms such as cell adhesion, leukocyte migration, and angiogenesis. Regarding cell composition (CC), enrichment areas included the extracellular space, extracellular exosome, extracellular region, and extracellular matrix. In terms of molecular function (MF), enrichment was found in integrin binding, heparin binding, calcium ion binding, and protein binding (**Figure 2A**). KEGG pathway analysis showed that the DEGs were mostly associated with the PPAR signaling pathway, regulation of lipolysis in adipocytes, ECM-receptor interaction, AMPK signaling pathway, and cell adhesion molecules (**Table 2**). These results indicate that most of the DEGs are significantly enriched in the extracellular matrix, cell cycle adhesion, and the PPAR signaling pathway. Chord plot depicting the relationship between genes and GO terms of the top 100 DEGs (**Figure 2B**). Gene set enrichment analysis (﻿GSEA) analysis ﻿revealed that genes involved in Ubiquitin mediated proteolysis and cell cycle were significantly enriched in MM compared to normal mesothelial tissues (**Figure 2C**).

**Table 2:** KEGG pathway enrichment analysis of DEGs.

Category	Term	Count	PValue	Genes
Upregulated genes	ECM-receptor interaction	5	4.67E-03	*COL1A1, COL3A1, ITGA3, HMMR, AGRN*
	Arrhythmogenic right ventricular cardiomyopathy	4	1.54E-02	*DSP, CDH2, ITGA3, PKP2*
	Biosynthesis of amino acids	4	1.86E-02	*PKM, TPI1, SHMT2, GAPDH*
	Protein processing in endoplasmic reticulum	5	4.30E-02	*SEC61A1, EIF2AK1, EDEM2, HYOU1, P4HB*
Downregulated genes	PPAR signaling pathway	11	2.08E-07	*FABP4, ACADL, ACSL1, SCD, ADIPOQ, LPL, PPARG, PLIN1, SORBS1, CD36, PCK1*
	Malaria	8	2.04E-05	*SELP, IL6, PECAM1, HBB, CD36, ACKR1, SELE, THBS4*
	Regulation of lipolysis in adipocytes	8	4.97E-05	*LIPE, FABP4, PDE3B, PTGER3, PLIN1, ADRB2, PIK3R1, PNPLA2*
	AMPK signaling pathway	11	5.49E-05	*LIPE, PFKFB3, SCD, LEP, ADIPOQ, PPARG, CD36, PIK3R1, PCK1, ACACB, FOXO1*
	Adipocytokine signaling pathway	7	1.37E-03	*SOCS3, ACSL1, LEP, ADIPOQ, CD36, PCK1, ACACB*

### PPI Network Construction and Module Analysis

**Figure 3:**
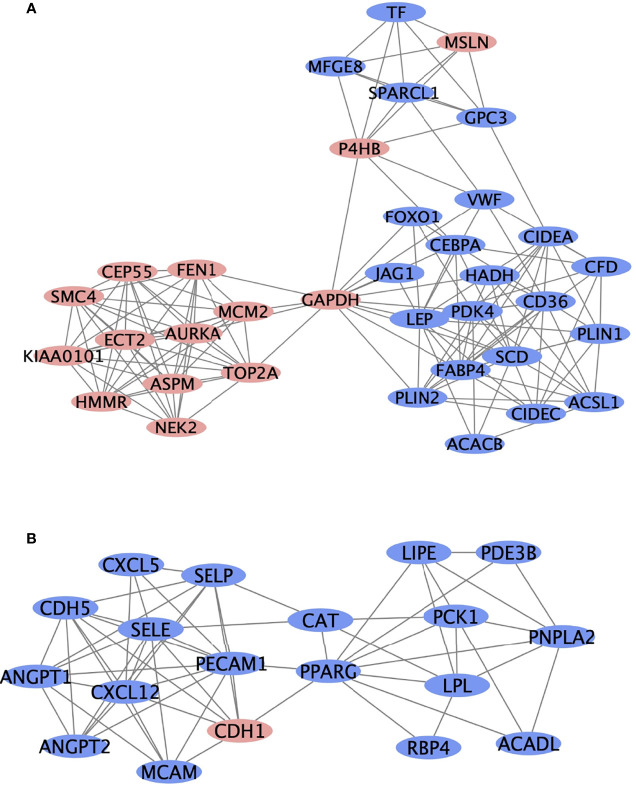
The top two most significant modules of DEGs. The top two significant modules **(A, B)** are selected from the PPI network. Red nodes represent upregulated genes, and blue nodes represent downregulated genes.

Protein interactions among a network of DEGs were constructed using STRING. A total of 305 nodes and 1217 edges were involved in the PPI network (**Figure 1D**) and the top 2 significant modules were obtained using Cytoscape (**Figure 3**). The functional analyses of genes involved in this module were analyzed using DAVID. Based on MCODE score, we identified two modules in the network with a setting k-score greater than 6. Module 1 included 35 nodes and 148 edges. Module 2 consisted of 19 nodes and 60 edges. The DEGs of modules 1 and 2 were significantly enriched in cell adhesion, angiogenesis, and protein phosphatase binding (**Table 3**). Cell adhesion molecules included *CD36, PECAM1, MSLN, MCAM, SELP, SELE*. Protein phosphatase binding included *CDH5, PPARG, NEK2* Angiogenesis included *JAG1, ANGPT2, ANGPT1, LEP, MCAM, PDE3B, PECAM1, MFGE8.*

**Table 3:** The Top 2 significantly modules.

Cluster	Score	Nodes	Edges	Node IDs
1	8.706	35	148	*VWF, LEP, ASPM, SPARCL1, AURKA, MCM2, KIAA0101, FEN1, TF, MFGE8, GPC3, SMC4, CFD, NEK2, HMMR, GAPDH, CEP55, ECT2, TOP2A, CEBPA, CIDEA, ACACB, PDK4, ACSL1, SCD, JAG1, PLIN2, CIDEC, HADH, MSLN, FABP4, P4HB, FOXO1, PLIN1, CD36*
2	6.667	19	60	*ACADL, LIPE, PPARG, PCK1, PNPLA2, ANGPT2, PECAM1, ANGPT1, CDH1, LPL, SELP, MCAM, SELE, CXCL5, CDH5, CXCL12, PDE3B, CAT, RBP4*

### Hub Gene Selection and Survival Analysis

**Figure 4:**
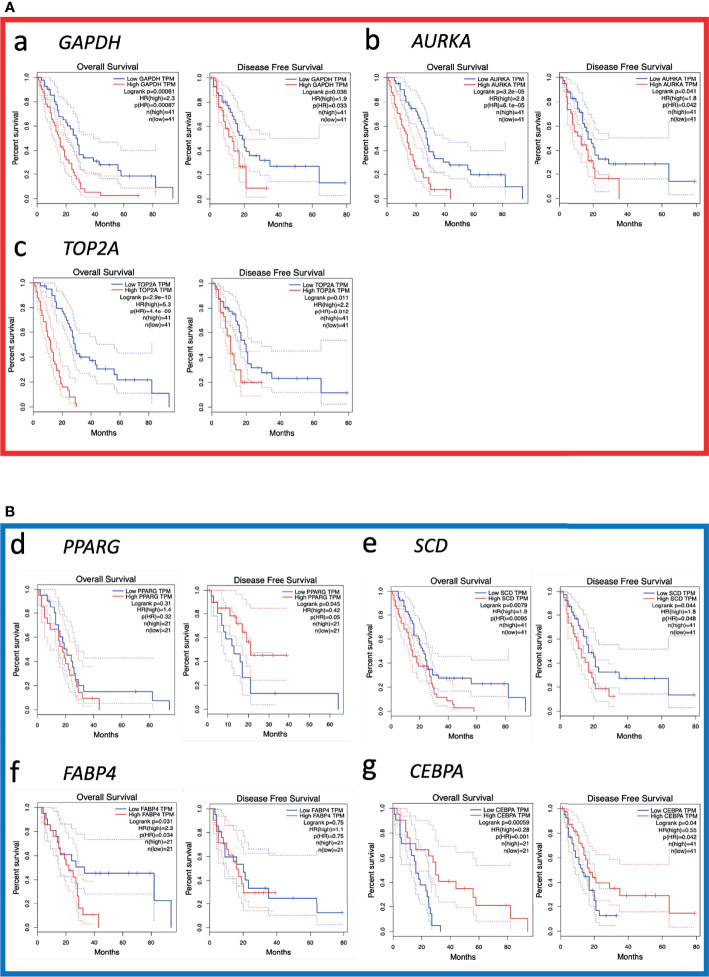
Kaplan-Meier survival analyses for the top 20 hub genes expressed in malignant mesothelioma patients. **(A)** Upregulated genes are significantly associated with overall survival (OS) and/or disease-free survival (DFS; red square). **(B)** Downregulated genes are significantly associated with OS and/or DFS (blue square). The other 13 genes without significant associations with OS and DFS are shown in **Supplemental Data**.

The top 20 genes of the PPI network were identified as hub genes with the Maximal Clique Centrality analysis method (**Table 4**). Subsequently, the overall survival analysis and disease-free survival analysis of the hub genes were performed using the GEPIA based on TCGA database. MM patients with higher expressions in *GAPDH*, *AURKA*, *TOP2A, SCD*, *and FABP4* showed worse overall survival than lower expressions (**Figure 4**). Nonetheless, MM patients with higher alterations in *GAPDH*, *AURKA*, *TOP2A*, and *SCD* showed worse disease-free survival than lower expressions (**Figure 4**). However, MM patients with higher *PPARG* expressions showed better disease-free survival. Moreover, high alteration of *CEBPA* was associated with more prolonged disease-free survival and better overall survival of MM (**Figure 4**). *GAPDH* has both glyceraldehyde-3-phosphate dehydrogenase and nitrosylase activities, thereby playing a role in glycolysis and nuclear functions, respectively. Aurora Kinase A encoded by *AURKA* is a mitotic serine/threonine kinase that contributes to the regulation of cell cycle progression. *TOP2A* encodes a DNA topoisomerase, an enzyme that controls and alters the topologic states of DNA during transcription. *SCD* encodes an enzyme involved in fatty acid biosynthesis, primarily the synthesis of oleic acid. *FABP4* roles include fatty acid uptake, transport, and metabolism. *PPARG* encodes a member of the peroxisome proliferator-activated receptor (PPAR) subfamily of nuclear receptors It’s a key regulator of adipocyte differentiation and glucose homeostasis. *CEBPA* gene encodes a transcription factor that contains a basic leucine zipper domain and recognizes the CCAAT motif inthe promoters of target genes. *CEBPA* coordinates proliferation arrest and the differentiation of myeloid progenitors, adipocytes, hepatocytes, and cells of the lung and the placenta

**Table 4:** Top 20 Hub gene in PPI network ranked by MCC method.

**Rank**	**Name**	**Score**	**Degree**
**1**	*PPARG*	3741564	39
**2**	*PNPLA2*	3726405	33
**3**	*LIPE*	3725389	26
**4**	*LPL*	3724044	28
**5**	*SCD*	3601850	24
**6**	*FABP4*	3443890	24
**7**	*LEP*	3128788	44
**8**	*PDK4*	2107470	19
**9**	*PLIN2*	1815265	16
**10**	*GAPDH*	1257609	83
**11**	*CIDEA*	1134028	16
**12**	*CIDEC*	1129272	15
**13**	*PCK1*	1020601	21
**14**	*PLIN1*	857570	18
**15**	*CD36*	766099	19
**16**	*ACSL1*	576013	18
**17**	*IL6*	491836	67
**18**	*CEBPA*	453645	17
**19**	*AURKA*	161314	15
**20**	*TOP2A*	161312	13

### Clinicopathological Characteristics

The demographic and follow-up information about the patients whose MM tissues were analyzed on the TMA are shown in **Table 5**. In total, 47 diagnosed cases of MM were analyzed: 44 in the peritoneum, 3 in the pleura. The mean age of the patients at diagnosis was 47 (range, 21-73) years; 18 patients were men, and 29 were women. Twenty-two of the 47 MM cases (20 in the peritoneum, 2 in the pleura) occurred in patients who were exposed to asbestos. The mean follow-up period ranged from 1 to 70 months. MM samples were subclassified as epithelioid (n = 42), biphasic (n = 3), or sarcomatoid (n = 2).

**Table 5:** Patient and tumor Characteristics of MM cases.

Characteristics	MM
Number of cases	47
Gender	18 M/29 F
Site	3 pleura/44 peritoneum
Age, median (range)	47 (21-73)
Histologic type	42 epithelioid/3 biphasic/2 sarcomatoid
Asbestos exposure (%)	22 (46.8%)
Alive with disease	4
Died of disease	43

### Immunohistochemistry

**Figure 5:**
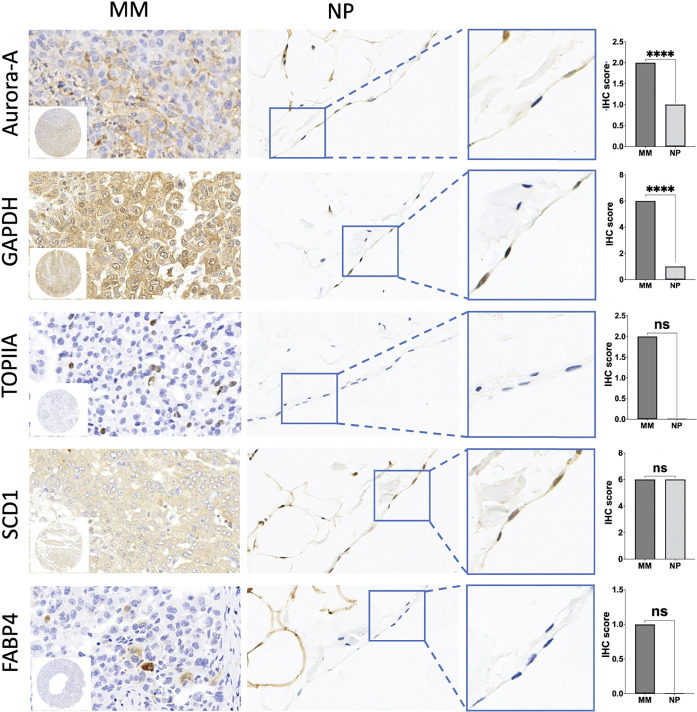
Immunohistochemical (IHC) staining of tissue microarrays (TMA) of malignant mesothelioma (MM) and normal peritoneal (NP) samples to identify the expression of hub genes. ﻿Amplification of IHC images of representative mesothelial cells in NP tissue was shown in the blue box. Significant differences are shown in Aurora-A and GAPDH. Quantification of IHC results of aforementioned key genes is shown in the box plot. ****p < 0.0001; ns, not statistically significant.

Immunohistochemistry was performed on the TMA with antibodies summarized in **Table 6**. The staining analysis is summarized in **Table 7**. GAPDH staining was primarily in the cytoplasm and found in 97% (44/47) of MM samples (**Figure 5**). The expression of GAPDH was significantly higher in MM than normal mesothelial cells (*p *< 0.0001). Additionally, GAPDH immunostaining was noted in some stromal fibroblasts in the TMA. Cytoplasm staining for GAPDH was present in 4 of 10 normal mesothelial tissue samples. Aurora-A membrane and cytoplasm staining was expressed in 76.7% (36/47) MM samples (**Figure 5**). Focal week cytoplasmic staining for Aurora-A was present in 2 of 10 normal mesothelial samples but was absent in all other NP cases. The expression of Aurora-A was significantly higher in MM samples than in NP samples (*p *< 0.0001). DNA topoisomerase II alpha (TOPIIA) expression was found in the nuclei of 19.1% (9/47) MM tissues but not in those of normal mesothelial samples; however, this difference was not statistically significant (**Figure 5**). SCD1 was expressed in the cytoplasm of 89.4% (42/47) MM tissues and 80% (8/10) NP tissues (*p *> 0.05); some stromal fibroblasts and endovascular cells expressed SCD1 as well. FABP4 was weakly expressed in the cytoplasm of 12.8% (6/47) MM tissues but no NP tissues (*p *> 0.05). Additionally, FABP4 immunostaining was noted in some fat stromal cells and endovascular cells in the TMA. All MM cases were negative for CEBPA and PPARG.

**Table 6:** Characteristics of antibodies used for IHC.

Protein (clone)	Antibody	Origin	Pretreatment	Dilution	Location of staining
SCD1(CD.E10)	Mouse mAb	Abcam	Dako 3 in 1 AR buffers EDTA pH 9.0	1:250	Cytoplasm
FABP4(EPR3579)	Rabbit mAb	Abcam	Tris/EDTA buffer, pH 9.0	1:10000	Cytoplasm
Topoisomerase II alpha (EP1102Y)	Rabbit mAb	Abcam	Tris/EDTA buffer, pH 9.0	1:5000	Nucleus
C/EBPα(D56F10)	Rabbit mAb	CST	Citrate Unmasking Solution	1:200	Cytoplasm
Phospho-Aurora-A (Thr288) (C39D8)	Rabbit mAb	CST	Citrate Unmasking Solution	1:800	Cytoplasm
Aurora-A(35C1)	Mouse mAb	Abcam	Tris/EDTA buffer, pH 9.0	1:200	Cytoplasm
PPARγ(E-8)	Mouse mAb	Santa Cruz Biotechnology	Citrate Unmasking Solution	1:250	Nucleus
GAPDH (14C10)	Rabbit mAb	CST	Citrate Unmasking Solution	1:800	Cytoplasm

mAb-monoclonal antibody.

**Table 7:** IHC staining in MM.

Specimens	Positive/Total, (%)						
	GAPDH	Aurora-A	TOPⅡA	SCD1	FABP4	CEBPA	PPARG
MM	44/47(93.6%)	36/47(75.6%)	9/47(19.1%)	42/47(89.4%)	6/47(12.8%)	0/47(0%)	0/47(0%)
Epithelioid	39/42(92.9%)	33/42(78.6%)	6/42(14.3%)	39/42(92.9%)	6/42(14.3%)	0/42(0%)	0/42(0%)
biphasic	3/3(100%)	2/3(66.7%)	2/3(66.7%)	2/3(66.7%)	0/3(0%)	0/3(0%)	0/3(0%)
sarcomatoid	2/2(100%)	1/2(50%)	1/2(50%)	1/2(50%)	0/2(0%)	0/2(0%)	0/2(0%)
normal peritoneum	4/10(40%)	2/10(20%)	0/10(0%)	8/10(80%)	0/10(0%)	0/10(0%)	0/10(0%)

**Figure 6:**
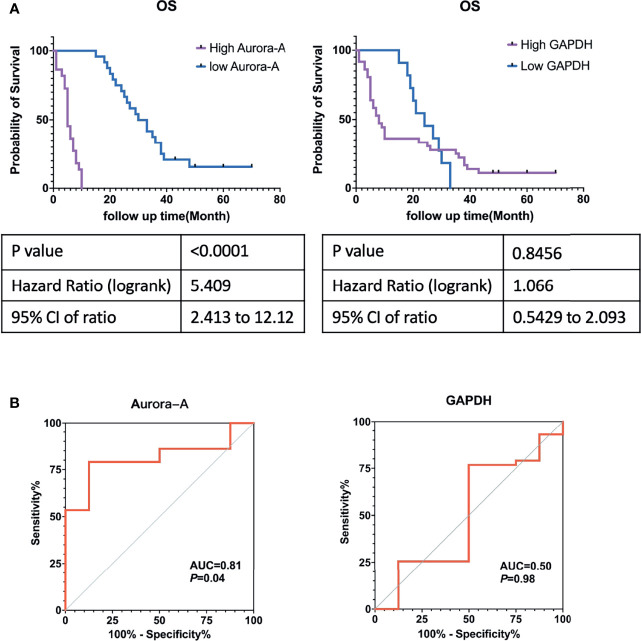
Aurora-A and GAPDH protein expression in tissue microarrays from patients with malignant mesothelioma. **(A)** Kaplan-Meier survival curves. **(B)** Receiver operating curves.

Kaplan-Meier analysis showed that MM cases with Aurora-A expression had significantly poorer overall survival rates (**Figure 6A**). The ROC curve of Aurora-A demonstrated diagnostic accuracy (area under the curve, 0.81; *p *= 0.04) (**Figure 6B**). However, GAPDH expression was not associated with overall survival and diagnostic accuracy in MM tissues (**Figures 6A, B**). The results suggested that Aurora-A could be an optimal diagnostic biomarker for identifying MM from benign mesothelial hyperplasia and it could be a potential prognostic marker for MM.

## Discussion

MM is a highly aggressive cancer with a poor prognosis for patients who receive multimodal treatment, including surgical resection, radiation therapy, chemotherapy, and immunotherapy (11, 12). Identifying molecular biomarkers is critically important for early diagnosis, prognosis prediction, and personalized therapy. Mesothelioma primarily arises from mesothelial cells in the thoracic and abdominal cavities. During embryogenesis, mesothelial cells are a single layer of mesodermal cells resting on a basement membrane that covers the celomic cavity. During the second month of human gestation, the celomic cavity is divided by the septum transversum into what will become the thoracic and abdominal cavities. Pleural and peritoneal mesothelioma has the same origin, similar histology and immunophenotypes. In this study, we analyzed the genes expression in GSE2549 and GSE112154 microarray datasets to identify common differentially expressed genes between pleural and peritoneal MM and verified them in TMA. To the best of our knowledge, this is the first report to demonstrate the differential expressed gene involvement of diagnostic-and prognosis-related genes in pleural and peritoneal MM.

A total of 346 DEGs were identified in the two GEO datasets, of which 111 genes were upregulated and 235 genes were downregulated. Subsequently, we utilized bioinformatics methods to deeply explore these DEGs, including GO term enrichment, signaling pathway enrichment, PPI network construction, hub genes selection, and survival analysis. Functional enrichment analysis of DEGs revealed primary involvement of cell adhesion, leukocyte migration, extracellular matrix, integrin binding, heparin binding, and calcium ion binding. KEGG pathway analysis showed that these genes were primarily associated with the PPAR signaling pathway, ECM-receptor interaction, regulation of lipolysis in adipocytes, AMPK signaling pathway, and cell adhesion molecules. ﻿GSEA analysis ﻿revealed that genes involved in Ubiquitin mediated proteolysis and cell cycle were significantly enriched in MM compared to normal mesothelial tissues

In our previous research, compared with Europe and the United States, the incidence of malignant mesothelioma in China has unique characteristics: 1) women have a high incidence; 2) peritoneal site is more common than pleural; 3) the age of onset is less than 50 years old (13, 14). In most industrialized countries, pleural MM characteristically occurs in a setting of asbestos exposure. Because men are most often involved in trades that expose them to asbestos, pleural MM is typically higher in men, with a male to female ratio of approximately 4:1 (15). However, among Chinese patients with pleural MM, 80% were women, and only 42% had been exposed to asbestos, underscoring the unusual prevalence of MM in Chinese women (13). These findings present a unique opportunity to investigate other causes of peritoneal MM in the Chinese population, aside from asbestos. In the United States and Europe, an increasing number of peritoneal MM cases do not seem to be associated with asbestos (16), leading some researchers to speculate that a subgroup of peritoneal MM cases may have a different pathogenesis.

Then, initial survival analysis was conducted with the gene expression profiles and clinical information from The Cancer Genome Atlas database of 87 patients who were diagnosed as having MM between 1999 and 2013. Seven key genes associated with prognosis—including *GAPDH*, *AURKA*, *TOP2A*, *SCD*, *FABP4*, *CEBPA*, and *PPARG*—were selected. To evaluate the diagnostic and prognostic value of seven key genes, we constructed a TMA consisting of 47 MM tissue samples and performed immunohistochemistry to verify the proteins encoded by those seven genes. GAPDH and Aurora-A expression were significantly higher in MM samples than in normal mesothelial samples (*p *< 0.0001). Kaplan-Meier analysis identified that only Aurora-A (encoded by *AURKA*) is associated with a poor prognosis.

As a pathologist, we identify malignant mesothelioma not only from other source cancer in pathological diagnostic work but also from benign mesothelial hyperplasia, especially in the case of pleural biopsies, abdominal biopsies, hydrothorax, and ascites specimens. How to distinguish between benign mesothelial hyperplasia and malignant mesothelioma has become particularly important. The commonly used mesothelioma markers such as Calretinin and WT-1 are not useful to identify mesothelioma from mesothelial hyperplasia. In this study, the expression of Aurora-A was significantly higher in MM samples than in Normal mesothelial samples. ROC curve analysis identified that Aurora-A has a diagnostic value. Aurora-A is a useful marker for identifying benign mesothelial hyperplasia and malignant mesothelioma.

Three members of the Aurora kinase family have been identified in mammals: Aurora-A, Aurora-B, and Aurora-C (17). Both Aurora-A and Aurora-B play essential roles in regulating cell division during mitosis, whereas Aurora-C has a unique physiological role in spermatogenesis. Aurora-A is a cell cycle-regulated serine/threonine-protein kinase that appears to be involved in crucial cancer-related signaling pathways such as the p53, Hippo, FOXO, PI3K-Akt, and Wnt pathways. Aurora-A has been a popular target for cancer therapy with nearly 50 clinical trials using specific Aurora-A kinase inhibitors (AKIs) (18). Aurora-A promotes tumorigenesis by participating in the cancer cell proliferation, apoptosis, epithelial-mesenchymal transition, self-renewal of cancer stem cells, and metastasis. Aurora-A interacts with numerous tumor suppressors and oncogenes, such as YBX1, Merlin, LDHB, ALDH1A1, Twist, YAP, GSK-3β, β-catenin, and ERα. In recent years, several selectively target Aurora-A small molecules have been identified with anticancer activity in preclinical studies, including BPR1K0609S1 (19, 20), LY3295668 (21), MK-8745 (22, 23), LDD970 (24), CYC3 (25), and AKI603 (26).

A phase 2 study for MLN8237 (alisertib), a selective Aurora-A kinase inhibitor, found an overall response rate of 27% in patients with relapsed and refractory aggressive B- and T-cell non-Hodgkin lymphomas (27). Alisertib seems to be clinically active in both B- and T-cell aggressive lymphomas. Based on these results, confirmatory single-agent and combination studies have been initiated. However, in an open-label, first-in-setting, randomized phase 3 trial to evaluate the efficacy of alisertib, in patients with relapsed/refractory peripheral T-cell lymphoma, alisertib was not significantly superior to the placebo comparator arm (28). A single-arm phase 2 study assessed single-agent efficacy and safety of alisertib in patients with platinum-refractory or -resistant epithelial ovarian, fallopian tube, or primary peritoneal carcinoma. Sixteen (52%) patients achieved stable disease with a mean response duration of 2.86 months, and 6 (19%) reached three or more months. Median progression-free survival and the time to progression were 1.9 months (29). In a multicenter phase 1/2 study, an objective response was noted in 9 (18%) of 49 women with breast cancer and 10 (21%) of 48 patients with small-cell lung cancer (30). In a phase 2 trial of patients with castration-resistant and neuroendocrine prostate cancer, a subset of patients with advanced prostate cancer and molecular features supporting Aurora-A and N-myc activation achieved significant clinical benefit from single-agent alisertib (31).

Alisertib plus cytarabine and idarubicin induction are effective and safe in acute myeloid leukemia patients (32, 33). 60 mg/m^2^ alisertib per dose for seven days shows antitumor activity, particularly in neuroblastoma patients with MYCN-nonamplified tumors (34, 35). EGFR-mutant lung adenocarcinoma cells with acquired resistance to third-generation EGFR inhibitors are sensitive to AKIs. Furthermore, combination treatment with AKIs and EGFR inhibitors was found to robustly decrease tumor growth in an EGFR-mutant lung adenocarcinoma PDX model (36). alisertib facilitates an anticancer immune microenvironment with decreased numbers of myeloid-derived suppressor cells and increased numbers of active CD8+ and CD4+ T lymphocytes (37). More importantly, the combined administration of alisertib and a PD-L1 antibody demonstrated synergistic efficacy for treating breast cancer cell 4T1 xenografts (37).

In this study, we identified common differentially expressed genes between pleural and peritoneal MM. Functional enrichment analysis of DEGs revealed primary involvement of cell adhesion, leukocyte migration, extracellular matrix, integrin binding, heparin binding, and calcium ion binding. KEGG pathway analysis showed that these genes were primarily associated with the PPAR signaling pathway, ECM-receptor interaction, regulation of lipolysis in adipocytes, and AMPK signaling pathway. Through TCGA database and immunochemistry analysis on Chinese MM TMA, Aurora-A expression was significantly higher in MM samples than in normal mesothelial samples (*p *< 0.0001). and Kaplan-Meier analysis and ROC curve analysis identified that only Aurora-A (encoded by *AURKA*) is associated with poor prognosis and has diagnostic value. At the same time, it may also be a potential therapeutic target.

## Conclusion

We identified Aurora-A as a prognostic and diagnostic marker for MM. This protein may predict the survival of patients with MM and play a role as a biomarker of diagnosis and sensitivity to target therapy. Moreover, we validated these results in tissue samples of patients with MM in China. However, due to the low incidence of mesothelioma, larger samples are needed to verify our results in the future. Taken together, Aurora-A shows promise as a diagnostic and prognostic marker for MM.

## Data Availability Statement

Publicly available datasets were analyzed in this study. This data can be found here: GSE2549 and GSE112154 in GEO database (https://www.ncbi.nlm.nih.gov/geo/).

## Ethics Statement

The studies involving human participants were reviewed and approved by The Institutional Review Board at the Cancer Hospital of the University of Chinese Academy of Sciences (Zhejiang Cancer Hospital). Written informed consent for participation was not required for this study in accordance with the national legislation and the institutional requirements.

## Author Contributions

ZGuo and YH guaranteed the manuscript’s integrity and were involved in the conceptualization and design of the study. ﻿ZGuo, LS, NL, and ZGu contributed to the data collection, analysis, and interpretation. ZGuo, XW, CW, ZC, JL, WM, and YH contributed to administrative, technical, or material support. All the authors contributed to writing and revising the manuscript. The final manuscript has been read and approved by all authors.

## Funding

This work was supported by grants from the National Natural Science Foundation of China (Grant No. 82072577), Natural Science Foundation of Zhejiang Province of China (Grant No. LY21H160002), Medical and Health Science and Technology Project of Zhejiang Province (No. 2020RC047), Key R&D Program Projects in Zhejiang Province (No. 2018C04009), National Natural Science Foundation of China (No .81672315), Clinical Research Fund of Zhejiang Medical Association (No. 2019ZYC-A77), International Cooperation Project of Zhejiang Basic Public Technology Research Program (No. LGJ20H010001), Projects of Zhejiang Province Medical and Health Science and Technology Plan (No. 2017KY256), Medical and Health Science and Technology Project of Zhejiang Province (No. 2021KY543), Medical and Health Science and Technology Plan Project of Zhejiang Province (No. 2019PY033), Research Program of Department of Science and Technology of Zhejiang Province (No. LGF19H180019), and Research Program of Zhejiang University 2018 Zhejiang Provincial Department of Education General Research Project (Natural Science) (No. Y201840845).

## Conflict of Interest

The authors declare that the research was conducted in the absence of any commercial or financial relationships that could be construed as a potential conflict of interest.

## Publisher’s Note

All claims expressed in this article are solely those of the authors and do not necessarily represent those of their affiliated organizations, or those of the publisher, the editors and the reviewers. Any product that may be evaluated in this article, or claim that may be made by its manufacturer, is not guaranteed or endorsed by the publisher.

## Supplementary Material

Supplementary Figure 1 Kaplan-Meier survival analyses for the top 20 hub genes expressed in malignant mesothelioma patients without statistical significance. **(A)** *PNPLA2*, **(B)** *LIPE*, **(C)** *LPL*, **(D)** *LEP*, **(E)** *PDK4*, **(F)** *PLIN2*, **(G)** *CIDEA*, **(H)** *CIDEC*, **(I)** *PCK1*, **(J)** *PLIN1*, **(K)** *CD36*, **(L)** *ACSL*, **(M)** *IL6*.

Supplementary Figure 2 IHC staining of PPARG and CEBPA in TMA of malignant mesothelioma (MM) and normal peritoneal (NP) samples. ﻿﻿Amplification of IHC images of representative mesothelial cells in NP tissue was shown in the blue box.
