# Supplementary figures and images for: Aurora Kinase A as a Diagnostic and Prognostic Marker of Malignant Mesothelioma
Source: Front Oncol. 2021 Dec 8;11:789244. doi: 10.3389/fonc.2021.789244 (PMC8692759; doi:10.3389/fonc.2021.789244)

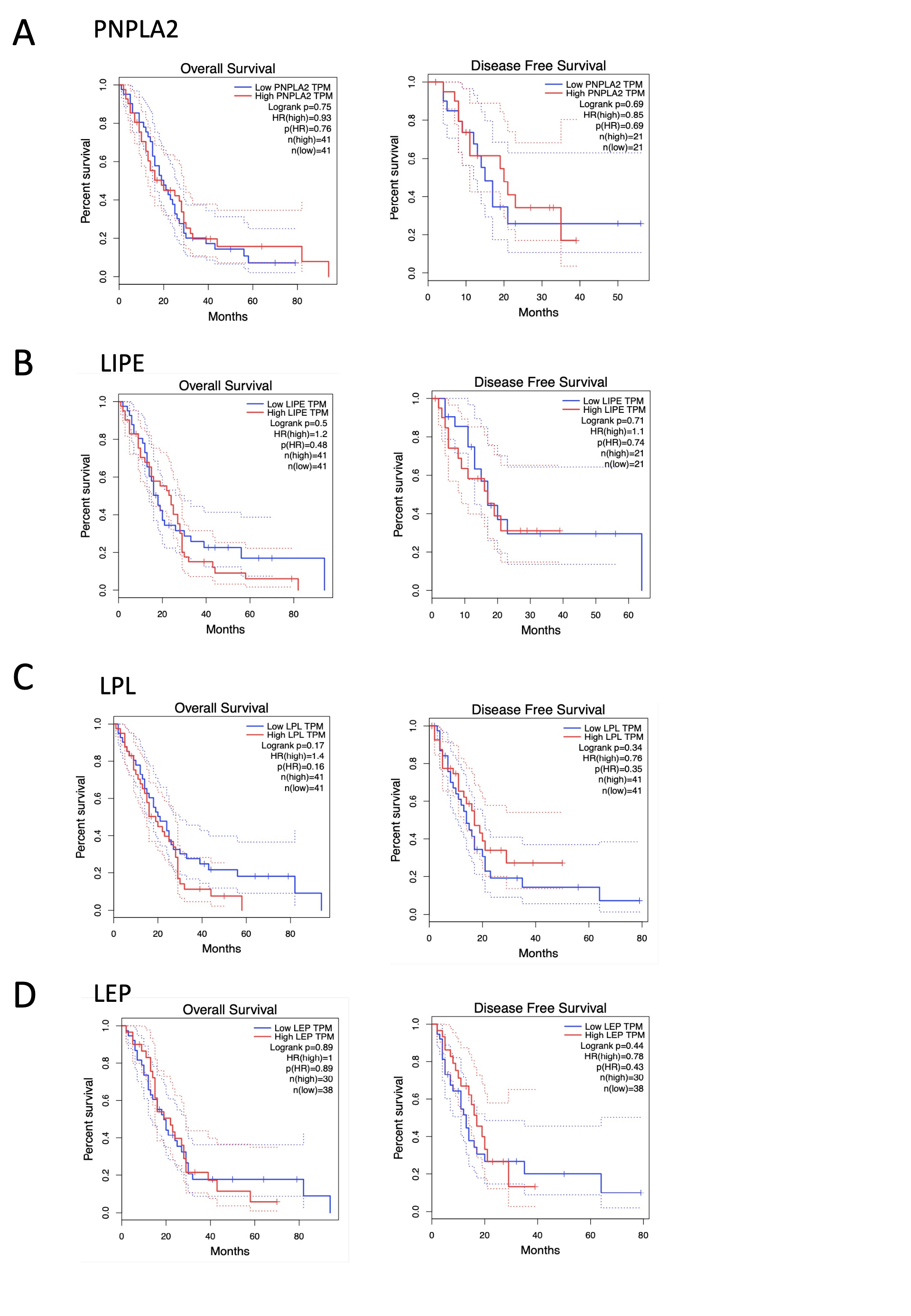

Supplement: Supplementary Figure 1 — Kaplan-Meier survival analyses for the top 20 hub genes expressed in malignant mesothelioma patients without statistical significance. (A) PNPLA2, (B) LIPE, (C) LPL, (D) LEP, (E) PDK4, (F) PLIN2, (G) CIDEA, (H) CIDEC, (I) PCK1, (J) PLIN1, (K) CD36, (L) ACSL, (M) IL6. [file DataSheet_1.zip › Supplementary image 1 A-D.JPEG]

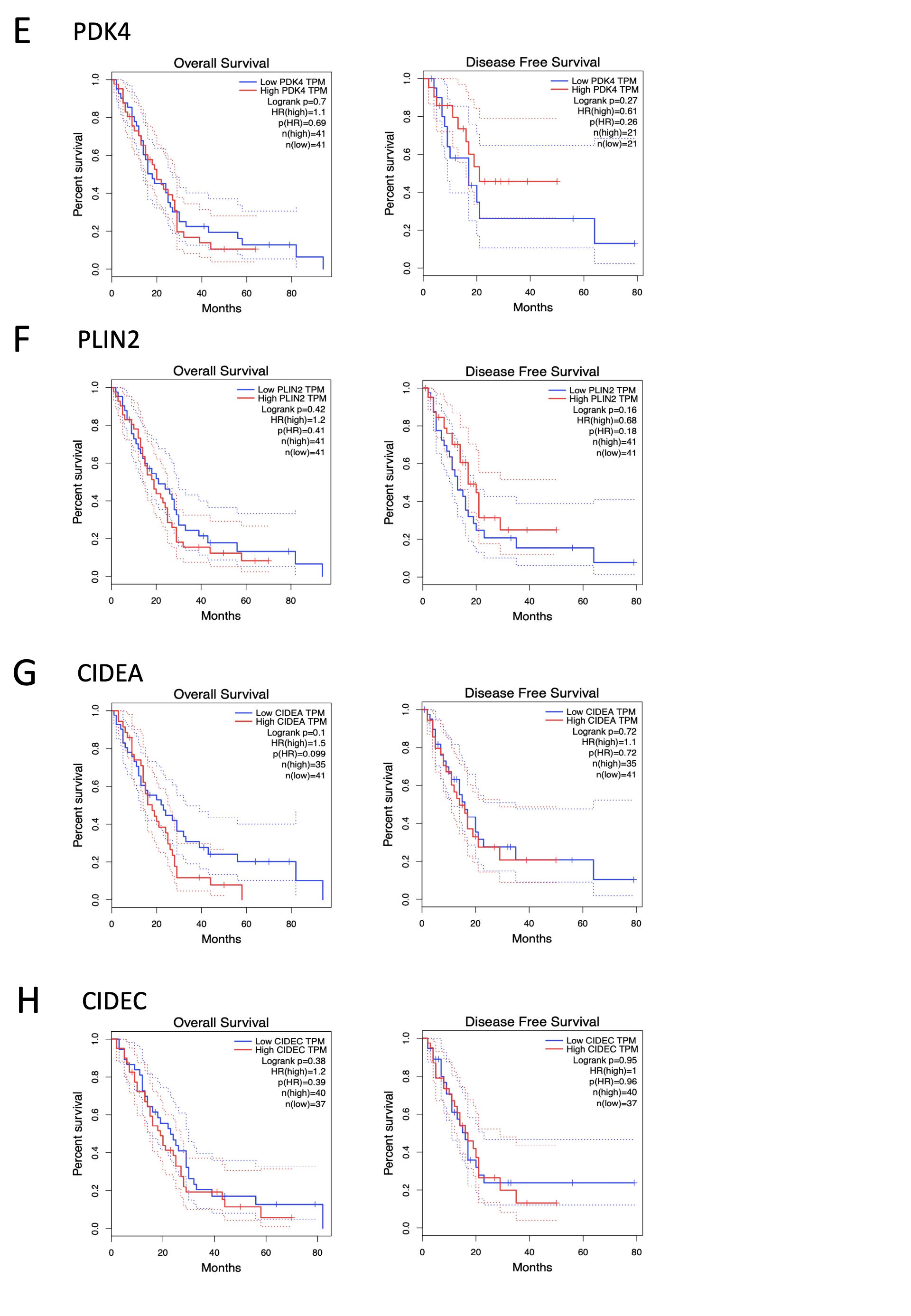

Supplement: Supplementary Figure 1 — Kaplan-Meier survival analyses for the top 20 hub genes expressed in malignant mesothelioma patients without statistical significance. (A) PNPLA2, (B) LIPE, (C) LPL, (D) LEP, (E) PDK4, (F) PLIN2, (G) CIDEA, (H) CIDEC, (I) PCK1, (J) PLIN1, (K) CD36, (L) ACSL, (M) IL6. [file DataSheet_1.zip › Supplementary image 1 E-H.JPEG]

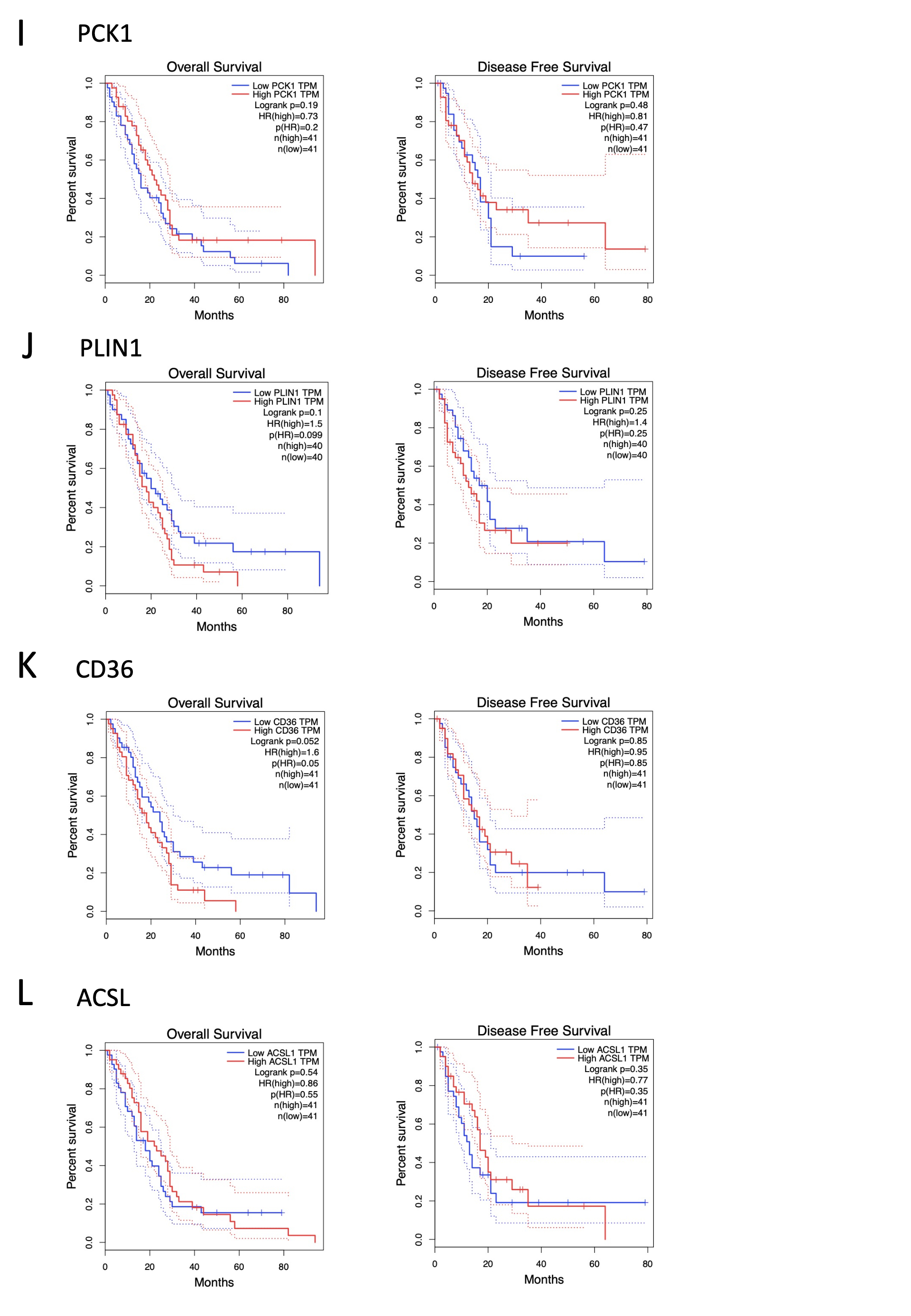

Supplement: Supplementary Figure 1 — Kaplan-Meier survival analyses for the top 20 hub genes expressed in malignant mesothelioma patients without statistical significance. (A) PNPLA2, (B) LIPE, (C) LPL, (D) LEP, (E) PDK4, (F) PLIN2, (G) CIDEA, (H) CIDEC, (I) PCK1, (J) PLIN1, (K) CD36, (L) ACSL, (M) IL6. [file DataSheet_1.zip › Supplementary image 1 I-L.JPEG]

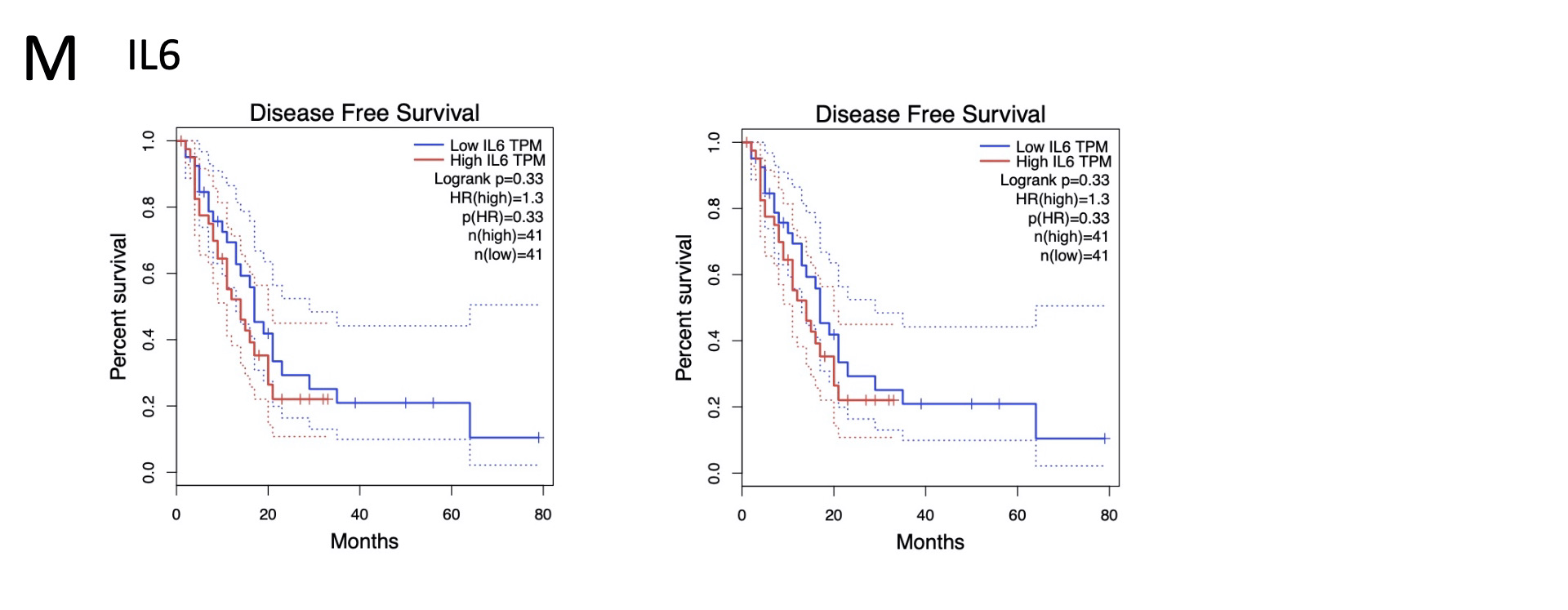

Supplement: Supplementary Figure 1 — Kaplan-Meier survival analyses for the top 20 hub genes expressed in malignant mesothelioma patients without statistical significance. (A) PNPLA2, (B) LIPE, (C) LPL, (D) LEP, (E) PDK4, (F) PLIN2, (G) CIDEA, (H) CIDEC, (I) PCK1, (J) PLIN1, (K) CD36, (L) ACSL, (M) IL6. [file DataSheet_1.zip › Supplementary image 1 M.JPEG]

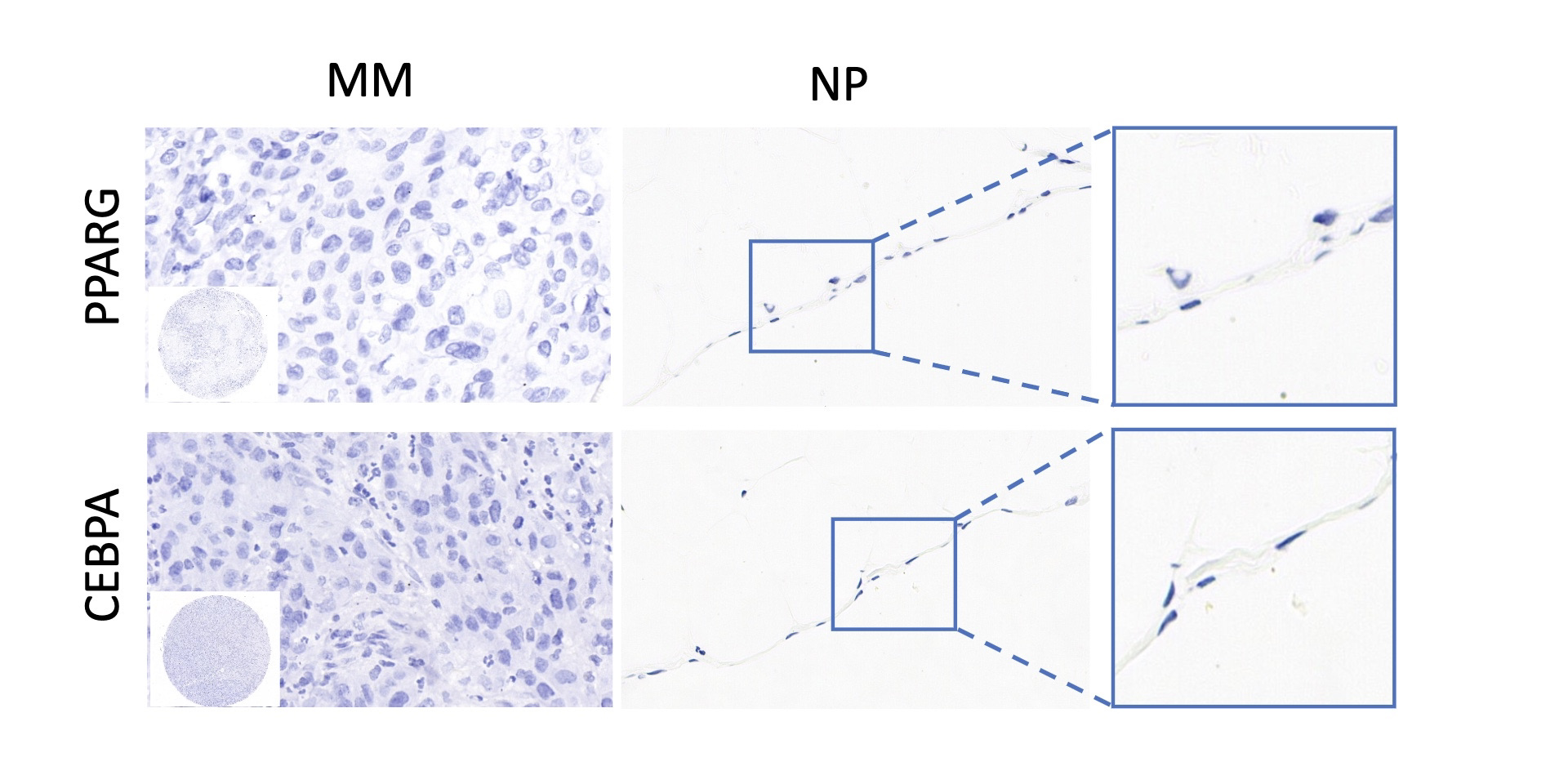

Supplement: Supplementary Figure 2 — IHC staining of PPARG and CEBPA in TMA of malignant mesothelioma (MM) and normal peritoneal (NP) samples. ﻿﻿Amplification of IHC images of representative mesothelial cells in NP tissue was shown in the blue box. [file Image_1.jpeg]
